# The complete moment convergence for CNA random vectors in Hilbert spaces

**DOI:** 10.1186/s13660-017-1566-x

**Published:** 2017-11-21

**Authors:** Mi-Hwa Ko

**Affiliations:** Division of Mathematics and Informational Statistics, Wonkwang University, Jeonbuk, 570-749 Korea

**Keywords:** 60F15, complete moment convergence, negative association, coordinatewise negatively associated random vectors, Hilbert spaces

## Abstract

In this paper we establish the complete moment convergence for sequences of coordinatewise negatively associated random vectors in Hilbert spaces. The result extends the complete moment convergence in (Ko in J. Inequal. Appl. 2016:131, 2016) to Hilbert spaces as well as generalizes the Baum-Katz type theorem in (Huan et al. in Acta Math. Hung. 144(1):132-149, 2014) to the complete moment convergence.

## Introduction

Ko et al. [3] introduced the concept of negative association (NA) for $\mathbb{R}^{d}$-valued random vectors. A finite family of $\mathbb{R}^{d}$-valued random vectors $\{X_{i}, 1\leq i \leq n\}$ is said to be negatively associated (NA) if for every pair of disjoint nonempty subsets *A* and *B* of $\{1,2,\ldots, n\}$ and any real coordinatewise nondecreasing functions *f* on $\mathbb{R}^{\vert A\vert d}$, *g* on $\mathbb{R}^{\vert B\vert d}$,

$$ \operatorname {Cov}\bigl(f(X_{i}, i\in A), g(X_{j}, j\in B) \bigr)\leq0, $$

whenever the covariance exists. Here and in the sequel, $\vert A\vert $ denotes the cardinality of *A*. An infinite family of $\mathbb{R}^{d}$-valued random vectors is NA if every finite subfamily is NA.

In the case $d=1$, the concept of negative association had already been introduced by Alam and Saxena [4] and carefully studied by Joag-Dev and Proschan [5].

A number of well-known multivariate distributions, such as a multinomial distribution, multivariate hypergeometric distribution, negatively correlated normal distribution and joint distribution of ranks, possess the NA property.

Let *H* be a real separable Hilbert space with the norm $\Vert \cdot \Vert $ generated by an inner product $\langle \cdot,\cdot\rangle $ and $\{e_{j}, j\geq1 \}$ be an orthonormal basis in *H*. Let *X* be an *H*-valued random vector and $\langle X, e_{j}\rangle $ be denoted by $X^{(j)}$.

Ko et al. [3] extended the concept of negative association in $\mathbb{R}^{d}$ to a Hilbert space as follows. A sequence $\{X_{n}, n \geq1\}$ of *H*-valued random vectors is said to be NA if for some orthonormal basis $\{e_{k}, k\geq1\}$ of *H* and for any $d\geq1$, the *d*-dimensional sequence $\{(X_{n}^{(1)}, X_{n}^{(2)},\ldots, X_{n} ^{(d)}), n\geq1\}$ of $\mathbb{R}^{d}$-valued random vectors is NA.

Ko et al. [3] proved almost sure convergence for *H*-valued NA random vectors and Thanh [6] proved almost sure convergence for *H*-valued NA random vectors and provided extensions of the results in Ko et al. [3]. Miao [7] showed Hajeck-Renyi inequality for NA random vectors in a Hilbert space.

Huan et al. [2] presented another concept of negative association for *H*-valued random vectors which is more general than the concept of *H*-valued NA random vectors introduced by Ko et al. [3] as follows.

A sequence $\{X_{n}, n\geq1\}$ of *H*-valued random vectors is said to be coordinatewise negatively associated (CNA) if, for each $j\geq1$, the sequence $\{X_{n}^{(j)}, n\geq1\}$ of random variables is NA, where $X_{n}^{(j)}=\langle X_{n}, e_{j}\rangle $.

Obviously, if a sequence of *H*-valued random vectors is NA, then it is CNA. However, the reverse is not true in general (see Example 1.4 of Huan et al. [2]).

Recently Huan et al. [2] showed Baum-Katz type theorems for CNA random vectors in Hilbert spaces and Huan [8] obtained the complete convergence for *H*-valued CNA random vectors with the *k*th partial sum. Hien and Thanh [9] investigated the weak laws of large numbers for sums of CNA random vectors in Hilbert spaces.

Let $\{X_{n}, n\geq1\}$ be a sequence of random variables. Let $\{a_{n}, n\geq1\}$ and $\{b_{n}, n\geq1\}$ be sequences of positive numbers and $q>0$. The concept of complete moment convergence is introduced as follows. If $\sum_{n=1}^{\infty}a_{n} E\{ b_{n}^{-1}\vert X _{n}\vert -\epsilon\}_{+}^{q}<\infty$ for all $\epsilon>0$, then $\{X_{n}, n\geq1\}$ is called the complete moment convergence.

Chow [10] first showed the complete moment convergence for a sequence of i.i.d. random variables by generalizing the result of Baum and Katz [11].

Since then, many complete moment convergences for various kinds of random variables in $\mathbb{R}^{1}$ have been established. For more details, we refer the readers to Liang and Li [12], Guo and Zhu [13], Wang and Hu [14], Wu et al. [15], Shen et al. [16], Wu and Jiang [17], and Ko [1] among others.

Let $\{X, X_{n}, n\geq1\}$ be a sequence of *H*-valued random vectors. We consider the following inequalities:

1.1 $$ C_{1} P \bigl( \bigl\vert X^{(j)} \bigr\vert >t \bigr) \leq \frac{1}{n}\sum_{k=1}^{n} P \bigl( \bigl\vert X_{k}^{(j)} \bigr\vert >t \bigr) \leq C_{2} P \bigl( \bigl\vert X^{(j)} \bigr\vert >t \bigr), $$

where $X_{n}^{(j)}=\langle X_{n}, e_{j}\rangle $ and $X^{(j)}=\langle X, e_{j}\rangle $ for all $j\geq1$.

If there exists a positive constant $C_{1}$ ($C_{2}$) such that the left-hand side (right-hand side) of (1.1) is satisfied for all $j\geq1$, $n\geq1$ and $t\geq0$, then the sequence $\{X_{n}, n\geq1 \}$ is said to be coordinatewise weakly lower (upper) bounded by *X*. The sequence $\{X_{n}, n\geq1\}$ is said to be coordinatewise weakly bounded by *X* if it is both coordinatewise weakly lower and upper bounded by *X*.

In this paper we show the complete moment convergence for CNA random vectors in Hilbert spaces. The result extends the complete moment convergence for NA random variables in $\mathbb{R}^{1}$ (the main result in Ko [1]) to a Hilbert space as well as generalizes the Baum-Katz type theorem (Theorem 2.1 in Huan et al. [2]) for CNA random vectors in a Hilbert space to the complete moment convergence in a Hilbert space.

## Preliminaries

The key tool for proving our results is the following maximal inequality.

### Lemma 2.1

(Huan et al. [2])

*Let* $\{X_{n}, n\geq1\}$ *be a sequence of* *H*-*valued CNA random vectors with* $EX_{n}=0$ *and* $E\Vert X_{n}\Vert ^{2}<\infty$ *for all* $n\geq1$. *Then we have*

2.1 $$ E\max_{1\leq k\leq n} \Biggl\Vert \sum_{i=1}^{k} X_{i} \Biggr\Vert ^{2}\leq2\sum _{i=1}^{n} E\Vert X_{i}\Vert ^{2},\quad n\geq1. $$

Taking $p=2$ in Lemma 3.1 of Huan et al. [2], we obtain the following lemma.

### Lemma 2.2

*Let* *r* *and* *α* *be positive real numbers such that* $1\leq r<2$ *and* $\alpha r>1$, *and let* *X* *be an* *H*-*valued random vector with*

2.2 $$ \sum_{j=1}^{\infty}E \bigl\vert X^{(j)} \bigr\vert ^{r}< \infty, $$

*where* $X^{(j)}=\langle X, e_{j}\rangle $. *Then we have*

2.3 $$ \sum_{j=1}^{\infty}\sum _{n=1}^{\infty}n^{\alpha (r-2)-1}E \bigl( \bigl(X^{(j)} \bigr)^{2}I \bigl( \bigl\vert X ^{(j)} \bigr\vert \leq n^{\alpha} \bigr) \bigr)< \infty. $$

### Remark

Let *X* be an *H*-valued random vector, where *H* is finite dimensional. If $E\Vert X\Vert ^{r}<\infty$, then

$$ \sum_{n=1}^{\infty}n^{\alpha(r-2)-1}E \bigl( \Vert X\Vert ^{2}I \bigl(\Vert X\Vert \leq n^{ \alpha} \bigr) \bigr)< \infty $$

holds.

### Lemma 2.3

(Kuczmaszewska [18])

*Let* $\{X_{n}, n\geq1\}$ *be a sequence of random variables weakly upper bounded by a random variable* *X*. *Let* $r>0$ *and*, *for some* $A>0$,

$$\begin{aligned}& X_{i}^{\prime}=X_{i} \bigl(\vert X_{i} \vert \leq A \bigr), \quad\quad X_{i}^{\prime\prime}=X_{i}I \bigl( \vert X_{i}\vert >A \bigr), \\& \tilde{X_{i}}=-AI(X_{i}< -A)+X_{i}I \bigl( \vert X_{i}\vert \leq A \bigr)+AI(X_{i}>A) \end{aligned}$$

*and*

$$\begin{aligned}& X^{\prime}=X \bigl(\vert X\vert \leq A \bigr), \quad\quad X^{\prime\prime }=XI \bigl(\vert X\vert >A \bigr), \\& \tilde{X}=-AI(X< -A)+XI \bigl(\vert X\vert \leq A \bigr)+AI(X>A). \end{aligned}$$

*Then*, *for some constant* $C>0$,

(i) *if* $E\vert X\vert ^{r}<\infty$, *then* $(n^{-1})\sum_{i=1}^{n} E\vert X_{i}\vert ^{r} \leq CE\vert X\vert ^{r}$,
(ii) $(n^{-1})\sum_{i=1}^{n} E\vert X_{i}^{\prime} \vert ^{r}\leq C(E\vert X^{\prime} \vert ^{r}+A ^{r} P(\vert X\vert >A))$ *for all* $A>0$,
(iii) $(n^{-1})\sum_{i=1}^{n} E\vert X_{i}^{\prime\prime} \vert ^{r}\leq CE\vert X^{\prime\prime} \vert ^{r}$ *for all* $A>0$,
(iv) $(n^{-1})\sum_{i=1}^{n} E\vert \tilde{X_{i}}\vert ^{r}\leq CE\vert \tilde{X}\vert ^{r}$ *for all* $A>0$.

The following result corresponds to Lemma 2.3 of Ko [1].

### Lemma 2.4

(Huan et al. [2])

*Let* *r* *and* *α* *be positive real numbers such that* $1\leq r<2$ *and* $\alpha r>1$, *and let* $\{X_{n}\}$ *be a sequence of* *H*-*valued CNA random vectors with zero means*. *If* $\{X_{n}, n\geq1\}$ *is coordinatewise weakly upper bounded by a random vector* *X* *satisfying* (2.2), *then*

2.4 $$ \sum_{n=1}^{\infty}n^{\alpha r-2} P \Biggl( \max_{1\leq k\leq n} \Biggl\Vert \sum_{i=1} ^{k} X_{i} \Biggr\Vert >\epsilon n^{\alpha} \Biggr)< \infty \quad {\textit{for every }}\epsilon >0. $$

The following lemma corresponds to Lemma 2.4 of Ko [1].

### Lemma 2.5

*Let* *r* *and* *α* *be positive real numbers such that* $1\leq r<2$ *and* $\alpha r>1$, *and let* $\{X_{n}, n\geq1\}$ *be a sequence of* *H*-*valued CNA random vectors with zero means*. *If* $\{X_{n}, n\geq1\}$ *is coordinatewise weakly upper bounded by a random vector* *X*, *then* (2.2) *implies*

2.5 $$ \sum_{n=1}^{\infty}n^{\alpha r-\alpha-2} \int_{n^{\alpha}}^{\infty }P \Biggl(\max_{1\leq k\leq n} \Biggl\Vert \sum_{i=1}^{k} X_{i} \Biggr\Vert >u \Biggr)\, du< \infty. $$

### Proof

For all $u>0$ and $j\geq1$, set

2.6 $$ Y_{ui}^{(j)}=X_{i}^{(j)}I \bigl( \bigl\vert X_{i}^{(j)} \bigr\vert \leq u \bigr)-uI \bigl(X_{i}^{(j)}< - u \bigr)+uI \bigl(X _{i}^{(j)}>u \bigr). $$

According to the proof of Theorem 2.1 in Huan et al. [2], we have

$$\begin{aligned}& \sum_{n=1}^{\infty}n^{\alpha r-2-\alpha} \int_{n^{\alpha}}^{ \infty}P \Biggl(\max_{1\leq k\leq n} \Biggl\Vert \sum_{i=1}^{k} X_{i} \Biggr\Vert >u \Biggr)\,du \\& \quad = \sum_{n=1}^{\infty}n^{\alpha r-2-\alpha} \int_{n^{\alpha}}^{ \infty}P \Biggl(\max_{1\leq k\leq n} \Biggl\Vert \sum_{i=1}^{k} \sum _{j=1}^{\infty}X _{i}^{(j)}e_{j} \Biggr\Vert >u \Biggr)\,du \\& \quad \leq \sum_{n=1}^{\infty}n^{\alpha r-2-\alpha} \int_{n^{\alpha}} ^{\infty}P \Bigl(\max_{1\leq k\leq n} \max_{j\geq1} \bigl\vert X_{k}^{(j)} \bigr\vert >u \Bigr) \,du \\& \quad \quad{} +\sum_{n=1}^{\infty}n^{\alpha r-2-\alpha} \int_{n^{\alpha}}^{\infty }P \Biggl(\max_{1\leq k\leq n} \Biggl\Vert \sum_{i=1}^{k} \sum _{j=1}^{\infty}Y_{ui} ^{(j)}e_{j} \Biggr\Vert >u \Biggr)\,du \\& \quad \leq \sum_{n=1}^{\infty}n^{\alpha r-2-\alpha} \int_{n^{\alpha}} ^{\infty}\sum_{j=1}^{\infty} \sum_{i=1}^{n} P \bigl( \bigl\vert X_{i}^{(j)} \bigr\vert >u \bigr) \,du \\& \quad \quad{} +\sum_{n=1}^{\infty}n^{\alpha r-2-\alpha} \int_{n^{\alpha}}^{\infty }P \Biggl(\max_{1\leq k\leq n} \Biggl\Vert \sum_{i=1}^{k} Y_{ui} \Biggr\Vert >u \Biggr)\,du \\& \quad =I_{1}+I_{2}. \end{aligned}$$

For $I_{1}$, by the Markov inequality, (1.1), (2.2) and the fact that $E\vert Y\vert ^{p}=p\int_{0}^{\infty}y^{p-1}P(\vert Y\vert >y)\,dy$, we obtain

2.7 $$\begin{aligned} I_{1} \leq&C\sum_{j=1}^{\infty}\sum _{n=1}^{\infty}n^{\alpha r-1- \alpha} \int_{n^{\alpha}}^{\infty}P \bigl( \bigl\vert X^{(j)} \bigr\vert >u \bigr)\,du \quad{ \bigl(\text{by (1.1)} \bigr)} \\ =&C\sum_{j=1}^{\infty} \int_{1}^{\infty}x^{\alpha r-1-\alpha} \int_{x^{\alpha}}^{\infty}P \bigl( \bigl\vert X^{(j)} \bigr\vert >u \bigr)\,du \, dx \quad \bigl(\text{letting }x^{ \alpha}=y \bigr) \\ =&C\sum_{j=1}^{\infty} \int_{1}^{\infty}y^{r-2} \int_{y}^{\infty }P \bigl( \bigl\vert X ^{(j)} \bigr\vert >u \bigr)\,du \,dy \\ =&C\sum_{j=1}^{\infty} \int_{1}^{\infty}P \bigl( \bigl\vert X^{(j)} \bigr\vert >u \bigr) \int_{1}^{u} y ^{r-2}\,dy \,du \\ \leq&C\sum_{j=1}^{\infty} \int_{0}^{\infty}u^{r-1}P \bigl( \bigl\vert X^{(j)} \bigr\vert >u \bigr)\,du \\ =&C\sum_{j=1}^{\infty}E \bigl\vert X^{(j)} \bigr\vert ^{r} \\ < &\infty\quad{ \bigl(\text{by (2.2)} \bigr)}. \end{aligned}$$

For $I_{2}$, we estimate that

$$\begin{aligned} I_{2} =&\sum_{n=1}^{\infty}n^{\alpha r-2-\alpha} \int_{n^{\alpha}} ^{\infty}P \Biggl(\max_{1\leq k\leq n} \Biggl\Vert \sum_{i=1}^{k} Y_{ui} \Biggr\Vert >u \Biggr)\,du \\ \leq&C\sum_{n=1}^{\infty}n^{\alpha r-2-\alpha} \int_{n^{\alpha}} ^{\infty}P \Biggl(\max_{1\leq k\leq n} \Biggl\Vert \sum_{i=1}^{k} Y_{ui}-EY_{ui} \Biggr\Vert > \frac{u}{2} \Biggr)\,du \\ &{}+C\sum_{n=1}^{\infty}n^{\alpha r-2-\alpha} \int_{n^{\alpha}}^{ \infty}P \Biggl(\max_{1\leq k\leq n} \Biggl\Vert \sum_{i=1}^{k} EY_{ui} \Biggr\Vert >\frac{u}{2} \Biggr)\,du \\ =&I_{21}+I_{22}. \end{aligned}$$

Since $\{Y_{ni}^{(j)}, i\geq1\}$ is NA for all $j\geq1$, and so $\{Y_{ni}, i\geq1\}$ is CNA. Hence, by the Markov inequality, Lemma 2.1 and Lemma 2.3(ii), we have

2.8 $$\begin{aligned} I_{21} \leq&C\sum_{n=1}^{\infty}n^{\alpha r-2-\alpha} \int_{n^{ \alpha}}^{\infty}u^{-2}E \Biggl(\max _{1\leq k\leq n} \Biggl\Vert \sum_{i=1}^{k} (Y _{ui}-EY_{ui}) \Biggr\Vert \Biggr)^{2}\,du \\ \leq&C\sum_{n=1}^{\infty}n^{\alpha r-2-\alpha} \int_{n^{\alpha}} ^{\infty}u^{-2}\sum _{i=1}^{n} E\Vert Y_{ui}-EY_{ui} \Vert ^{2}\,du \quad{\text{by (2.1)}} \\ \leq&C\sum_{n=1}^{\infty}n^{\alpha r-2-\alpha} \int_{n^{\alpha}} ^{\infty}u^{-2}\sum _{i=1}^{n} E\Vert Y_{ui}\Vert ^{2}\,du \\ \leq&C\sum_{j=1}^{\infty}\sum _{n=1}^{\infty}n^{\alpha r-2-\alpha } \int_{n^{\alpha}}^{\infty}u^{-2}\sum _{i=1}^{n}E \bigl(Y_{ui}^{(j)} \bigr)^{2}\,du \\ \leq&C\sum_{j=1}^{\infty}\sum _{n=1}^{\infty}n^{\alpha r-1-\alpha } \int_{n^{\alpha}}^{\infty}P \bigl( \bigl\vert X^{(j)} \bigr\vert >u \bigr)\,du \\ &{}+C\sum_{j=1}^{\infty}\sum _{n=1}^{\infty}n^{\alpha r-1-\alpha} \int_{n^{\alpha}}^{\infty}u^{-2}E \bigl( \bigl\vert X^{(j)} \bigr\vert ^{2}I \bigl( \bigl\vert X^{(j)} \bigr\vert \leq u \bigr) \bigr)\,du \\ =&I_{211}+I_{212}. \end{aligned}$$

The last inequality above is obtained by Lemma 2.3(ii).

For $I_{211}$, by (2.7) we have that $I_{211}< \infty$.

For $I_{212}$, by a standard calculation we observe that

2.9 $$\begin{aligned} I_{212} =&C\sum_{n=1}^{\infty}n^{\alpha r-1-\alpha} \sum_{j=1}^{ \infty}\sum _{m=n}^{\infty} \int_{m^{\alpha}}^{(m+1)^{\alpha}} u ^{-2}E \bigl( \bigl(X^{(j)} \bigr)^{2}I \bigl( \bigl\vert X^{(j)} \bigr\vert \leq u \bigr) \bigr)\,du \\ \leq&C\sum_{n=1}^{\infty}n^{\alpha r-1-\alpha}\sum _{j=1}^{\infty }\sum_{m=n}^{\infty}m^{-\alpha-1}E \bigl( \bigl(X^{(j)} \bigr)^{2}I \bigl( \bigl\vert X^{(j)} \bigr\vert \leq(m+1)^{ \alpha} \bigr) \bigr) \\ =&C\sum_{j=1}^{\infty}\sum _{m=1}^{\infty}m^{-\alpha-1}E \bigl( \bigl\vert X^{(j)} \bigr\vert ^{2}I \bigl( \bigl\vert X ^{(j)} \bigr\vert \leq(m+1)^{\alpha} \bigr) \bigr)\sum _{n=1}^{m} n^{\alpha r-1-\alpha} \\ \leq&C\sum_{j=1}^{\infty}\sum _{m=1}^{\infty}m^{\alpha r-2\alpha-1} \sum _{n=1}^{m+1}E \bigl( \bigl\vert X^{(j)} \bigr\vert ^{2}I \bigl((n-1)^{\alpha }< \bigl\vert X^{(j)} \bigr\vert \leq n^{ \alpha} \bigr) \bigr) \\ \leq&C\sum_{j=1}^{\infty}\sum _{m=1}^{\infty}m^{\alpha r}P \bigl((m-1)^{ \alpha}< \bigl\vert X^{(j)} \bigr\vert \leq m^{\alpha} \bigr)) \\ \leq&C\sum_{j=1}^{\infty}E \bigl\vert X^{(j)} \bigr\vert ^{r}< \infty. \end{aligned}$$

It remains to prove $I_{22}<\infty$. From (1.1), (2.2), (2.6) and the fact that $EX_{i}^{(j)}=0$, for all $i\geq1$ and $j\geq1$, we obtain

2.10 $$\begin{aligned} I_{22} =&C\sum_{n=1}^{\infty}n^{\alpha r-2-\alpha} \int_{n^{\alpha}} ^{\infty}P \Biggl(\max_{1\leq k\leq n} \Biggl\Vert \sum_{i=1}^{k} EY_{ui} \Biggr\Vert > \frac{u}{2} \Biggr)\,du \\ \leq&C\sum_{n=1}^{\infty}n^{\alpha r-2-\alpha} \int_{n^{\alpha}} ^{\infty}P \Biggl(\sum _{i=1}^{n} E\Vert Y_{ui}\Vert > \frac{u}{2} \Biggr)\,du \\ \leq&C\sum_{n=1}^{\infty}n^{\alpha r-2-\alpha} \int_{n^{\alpha}} ^{\infty}u^{-1}\sum _{i=1}^{n} E\Vert Y_{ui}\Vert \,du \\ \leq&C\sum_{j=1}^{\infty}\sum _{n=1}^{\infty}n^{\alpha r-1-\alpha } \int_{n^{\alpha}}^{\infty} \bigl( P \bigl( \bigl\vert X^{(j)} \bigr\vert >u \bigr) \bigr)\,du \\ &{} +C\sum_{j=1}^{\infty}\sum _{n=1}^{\infty}n^{\alpha r-1-\alpha} \int_{n^{\alpha}}^{\infty}u^{-1} E \bigl( \bigl\vert X^{(j)} \bigr\vert I \bigl( \bigl\vert X^{(j)} \bigr\vert \leq u \bigr) \bigr)\,du \\ =&I_{221}+I_{222}. \end{aligned}$$

By (2.7) we have that $I_{221}<\infty$.

For $I_{222}$, by a standard calculation as in (2.9), we obtain

$$\begin{aligned} I_{222} =&C\sum_{j=1}^{\infty}\sum _{n=1}^{\infty}n^{\alpha r-1- \alpha} \int_{n^{\alpha}}^{\infty}u^{-1} E \bigl( \bigl\vert X^{(j)} \bigr\vert I \bigl( \bigl\vert X^{(j)} \bigr\vert \leq u \bigr) \bigr)\,du \\ \leq&C\sum_{j=1}^{\infty}\sum _{n=1}^{\infty}n^{\alpha r-1-2\alpha }\sum _{m=n}^{\infty} \int_{m^{\alpha}}^{(m+1)^{\alpha}} E \bigl( \bigl\vert X^{(j)} \bigr\vert I \bigl( \bigl\vert X ^{(j)} \bigr\vert \leq(m+1)^{\alpha} \bigr) \bigr)\,du \\ =&C\sum_{j=1}^{\infty}\sum _{n=1}^{\infty}n^{\alpha r-1-2\alpha} \sum _{m=n}^{\infty} \bigl((m+1)^{\alpha}-m^{\alpha} \bigr) E \bigl( \bigl\vert X^{(j)} \bigr\vert I \bigl( \bigl\vert X^{(j)} \bigr\vert \leq(m+1)^{\alpha} \bigr) \bigr) \\ \leq&C\sum_{j=1}^{\infty}\sum _{n=1}^{\infty}n^{\alpha r-1-2\alpha }\sum _{m=1}^{\infty}m^{\alpha-1} E \bigl( \bigl\vert X^{(j)} \bigr\vert I \bigl( \bigl\vert X^{(j)} \bigr\vert \leq(m+1)^{ \alpha} \bigr) \bigr) \\ \leq&C\sum_{j=1}^{\infty}\sum _{m=1}^{\infty}m^{\alpha-1} E \bigl( \bigl\vert X ^{(j)}\vert I \bigl(\vert X^{(j)} \bigr\vert \leq(m+1)^{\alpha } \bigr) \bigr)\sum_{n=1}^{m+1} n^{\alpha r-1-2 \alpha} \\ \leq&C\sum_{j=1}^{\infty}\sum _{m=1}^{\infty}m^{\alpha r-1-\alpha } \sum _{n=1}^{m+1} E \bigl( \bigl\vert X^{(j)} \bigr\vert I \bigl((n-1)^{\alpha}< \bigl\vert X^{(j)} \bigr\vert \leq n^{ \alpha} \bigr) \bigr) \\ \leq&C\sum_{j=1}^{\infty}\sum _{m=1}^{\infty}m^{\alpha r} P \bigl((m-1)^{ \alpha}< \bigl\vert X^{(j)} \bigr\vert \leq m^{\alpha} \bigr) \\ \leq&C\sum_{j=1}^{\infty}E \bigl\vert X^{(j)} \bigr\vert ^{r}< \infty, \end{aligned}$$

which yields $I_{22}<\infty$, together with $I_{221}<\infty$. Hence, the proof is completed. □

The following lemma shows that Lemmas 2.4 and 2.5 still hold under a sequence of identically distributed *H*-valued CNA random vectors with zero means.

### Lemma 2.6

*Let* *r* *and* *α* *be positive real numbers such that* $1\leq r<2$ *and* $\alpha r > 1$, *and let* $\{X_{n}, n\geq1\}$ *be a sequence of* *H*-*valued CNA random vectors with zero means*. *If* $\{X_{n}, n\geq1\}$ *are identically distributed random vectors with*

2.2′ $$ \sum_{j=1}^{\infty}E \bigl\vert X_{1}^{(j)} \bigr\vert ^{r}< \infty, $$

*where* $X_{1}^{(j)}=\langle X_{1}, e_{j}\rangle $, *then* (2.4) *and* (2.5) *hold*.

### Proof

The proofs are similar to those of Lemma 2.4 and Lemma 2.5, respectively. □

### Lemma 2.7

(Huan et al. [2])

*Let* *r* *and* *α* *be positive real numbers such that* $\alpha r\geq1$, *and let* $\{X_{n}, n \geq1\}$ *be a sequence of* *H*-*valued CNA random vectors with zero means*. *Suppose that* $\{X_{n}, n\geq1\}$ *is coordinatewise weakly bounded by a random vector* *X* *with*

2.11 $$ \sum_{j=1}^{\infty}E \bigl( \bigl\vert X^{(j)} \bigr\vert ^{r}I \bigl( \bigl\vert X^{(j)} \bigr\vert \leq1 \bigr) \bigr)< \infty. $$

*If*

2.12 $$ \sum_{j=1}^{\infty}\sum _{n=1}^{\infty}n^{\alpha r-2}P \Biggl( \max _{1\leq k\leq n} \Biggl\vert \sum_{l=1}^{k} X_{l}^{(j)} \Biggr\vert >\epsilon n^{\alpha} \Biggr)< \infty \quad {\textit{for every }}\epsilon>0, $$

*then* (2.2) *holds*.

### Proof

See the proof of Theorem 2.6 in Huan et al. [2]. □

The following section will show that the complete moment convergence for NA random variables in Ko [1] can be extended to a Hilbert space.

## Main results

The proofs of main results can be obtained by using the methods of the proofs as in the main results of Ko [1].

### Theorem 3.1

*Let* *r* *and* *α* *be positive numbers such that* $1\leq r<2$ *and* $\alpha r>1$. *Let* $\{X_{n}, n\geq1\}$ *be a sequence of* *H*-*valued CNA random vectors with zero means*. *If* $\{X_{n}, n\geq1\}$ *is coordinatewise weakly upper bounded by a random vector* *X* *satisfying* (2.2), *then we obtain*

3.1 $$ \sum_{n=1}^{\infty}n^{\alpha r-2-\alpha} E \Biggl( \max_{1\leq k\leq n} \Biggl\Vert \sum_{i=1}^{k} X_{i} \Biggr\Vert -\epsilon n^{\alpha} \Biggr)^{+}< \infty, $$

*where* $a^{+}=\max\{a,0\}$.

### Proof

The proof can be obtained by a similar calculation in the proof of Theorem 3.1 of Ko [1]. From Lemmas 2.4 and 2.5 we obtain

3.2 $$\begin{aligned}& \sum_{n=1}^{\infty}n^{\alpha r-2-\alpha}E \Biggl( \max_{1\leq k\leq n} \Biggl\Vert \sum_{i=1}^{k} X_{i} \Biggr\Vert -\epsilon n^{\alpha} \Biggr)^{+} \\& \quad = \sum_{n=1}^{\infty}n^{\alpha r-2-\alpha} \int_{0}^{\infty}P \Biggl( \Biggl( \max _{1\leq k\leq n} \Biggl\Vert \sum_{i=1}^{k} X_{i} \Biggr\Vert -\epsilon n^{\alpha} \Biggr)^{+}>u \Biggr)\,du \\& \quad = \sum_{n=1}^{\infty}n^{\alpha r-2-\alpha} \int_{0}^{\infty}P \Biggl( \max_{1\leq k\leq n} \Biggl\Vert \sum_{i=1}^{k} X_{i} \Biggr\Vert -\epsilon n^{\alpha}>u \Biggr)\,du \\& \quad = \sum_{n=1}^{\infty}n^{\alpha r-2-\alpha} \int_{0}^{n^{\alpha}} P \Biggl( \max_{1\leq k\leq n} \Biggl\Vert \sum_{i=1}^{k} X_{i} \Biggr\Vert -\epsilon n^{\alpha}>u \Biggr)\,du \\& \quad \quad{} +\sum_{n=1}^{\infty}n^{\alpha r-2-\alpha} \int_{n^{\alpha}}^{\infty }P \Biggl(\max_{1\leq k\leq n} \Biggl\Vert \sum_{i=1}^{k} X_{i} \Biggr\Vert -\epsilon n^{\alpha}>u \Biggr)\,du \\& \quad \leq \sum_{n=1}^{\infty}n^{\alpha r-2} P \Biggl(\max_{1\leq k\leq n} \Biggl\Vert \sum _{i=1}^{k} X_{i} \Biggr\Vert >\epsilon n^{\alpha} \Biggr) \\& \quad \quad{} +\sum_{n=1}^{\infty}n^{\alpha r-2-\alpha} \int_{n^{\alpha}}^{\infty }P \Biggl(\max_{1\leq k\leq n} \Biggl\Vert \sum_{i=1}^{k} X_{i} \Biggr\Vert >u \Biggr)\,du \\& \quad < \infty. \end{aligned}$$

 □

### Theorem 3.2

*Let* *r* *and* *α* *be positive numbers such that* $1\leq r<2$, $\alpha r> 1$ *and* $\alpha>\frac{1}{2}$. *Let* $\{X_{n}, n\geq1\}$ *be a sequence of* *H*-*valued CNA random vectors with zero means*. *If* $\{X_{n}, n\geq1\}$ *is coordinatewise weakly upper bounded by a random vector* *X*, *then* (3.1) *implies* (2.4).

### Proof

It follows from (3.2) that

3.3 $$\begin{aligned}& \sum_{n=1}^{\infty}n^{\alpha r-2-\alpha}E \Biggl( \max_{1\leq k\leq n} \Biggl\Vert \sum_{i=1}^{k} X_{i} \Biggr\Vert -\epsilon n^{\alpha} \Biggr)^{+} \\& \quad = \sum_{n=1}^{\infty}n^{\alpha r-2-\alpha} \int_{0}^{\infty}P \Biggl( \max_{1\leq k\leq n} \Biggl\Vert \sum_{i=1}^{k} X_{i} \Biggr\Vert -\epsilon n^{\alpha}>u \Biggr)\,du \\& \quad \geq \sum_{n=1}^{\infty}n^{\alpha r-2-\alpha} \int_{0}^{\epsilon n ^{\alpha}} P \Biggl(\max_{1\leq k\leq n} \Biggl\Vert \sum_{i=1}^{k} X_{i} \Biggr\Vert >\epsilon n^{\alpha}+u \Biggr)\,du \\& \quad \geq \epsilon\sum_{n=1}^{\infty}n^{\alpha r-2} P \Biggl(\max_{1\leq k \leq n} \Biggl\Vert \sum _{i=1}^{k} X_{i} \Biggr\Vert >2\epsilon n^{\alpha} \Biggr). \end{aligned}$$

Hence, (3.3) and (3.1) imply (2.4). The proof Theorem 3.2 is complete. □

### Theorem 3.3

*Let* *r* *and* *α* *be positive numbers such that* $1\leq r<2$, $\alpha r> 1$ *and* $\alpha>\frac{1}{2}$. *Let* $\{X_{n}, n\geq1\}$ *be a sequence of* *H*-*valued CNA random vectors with zero means*. *If* $\{X_{n}, n\geq1\}$ *is coordinatewise weakly upper bounded by a random vector* *X*, *then* (2.2) *implies*

3.4 $$ \sum_{n=1}^{\infty}n^{\alpha r-2}E \Biggl\{ \sup_{k\geq n}k^{-\alpha} \Biggl\Vert \sum _{i=1}^{k} X_{i} \Biggr\Vert -\epsilon \Biggr\} ^{+}< \infty. $$

### Proof

(3.1) provides that

$$\begin{aligned}& \sum_{n=1}^{\infty}n^{\alpha r-2}E \Biggl\{ \sup_{k\geq n}k^{-\alpha} \Biggl\Vert \sum _{i=1}^{k} X_{i} \Biggr\Vert -\epsilon \Biggr\} ^{+} \\& \quad = \sum_{n=1}^{\infty}n^{\alpha r-2} \int_{0}^{\infty}P \Biggl(\sup_{k \geq n} k^{-\alpha} \Biggl\Vert \sum_{i=1}^{k} X_{i} \Biggr\Vert >\epsilon+u \Biggr)\,du \\& \quad = \sum_{m=1}^{\infty}\sum _{n=2^{m-1}}^{2^{m}-1}n^{\alpha r-2} \int _{0}^{\infty}P \Biggl(\sup_{k\geq n} k^{-\alpha} \Biggl\Vert \sum_{i=1}^{k} X_{i} \Biggr\Vert > \epsilon+u \Biggr)\,du \\& \quad \leq C\sum_{m=1}^{\infty} \int_{0}^{\infty}P \Biggl(\sup_{k\geq2^{m-1}} k ^{-\alpha} \Biggl\Vert \sum_{i=1}^{k} X_{i} \Biggr\Vert >\epsilon+u \Biggr)\,du \sum _{n=2^{m-1}} ^{2^{m}-1}n^{\alpha r-2} \\& \quad \leq C\sum_{m=1}^{\infty}2^{m(\alpha r-1)} \int_{0}^{\infty}P \Biggl( \sup_{k\geq2^{m-1}} k^{-\alpha} \Biggl\Vert \sum_{i=1}^{k} X_{i} \Biggr\Vert >\epsilon+u \Biggr)\,du \\& \quad \leq C\sum_{m=1}^{\infty}2^{m(\alpha r-1)}\sum _{l=m}^{\infty} \int _{0}^{\infty}P \Biggl(\max_{2^{l-1}\leq k< 2^{l}} k^{-\alpha} \Biggl\Vert \sum_{i=1} ^{k} X_{i} \Biggr\Vert >\epsilon+u \Biggr)\,du \\& \quad = C\sum_{l=1}^{\infty} \int_{0}^{\infty}P \Biggl(\max_{2^{l-1}\leq k\leq2^{l}} k^{-\alpha} \Biggl\Vert \sum_{i=1}^{k} X_{i} \Biggr\Vert >\epsilon+u \Biggr)\, du \sum _{m=1}^{l} 2^{m( \alpha r-1)} \\& \quad \leq C\sum_{l=1}^{\infty}2^{l(\alpha r-1)} \int_{0}^{\infty}P \Biggl( \max_{2^{l-1}\leq k\leq2^{l}} \Biggl\Vert \sum_{i=1}^{k} X_{i} \Biggr\Vert >(\epsilon+u)2^{(l-1) \alpha} \Biggr)\,du \\& \quad \quad \bigl(\text{letting } y=2^{(l-1)\alpha}u \bigr) \\& \quad \leq C\sum_{l=1}^{\infty}2^{l(\alpha r-1-\alpha)} \int_{0}^{\infty }P \Biggl(\max_{1\leq k\leq2^{l}} \Biggl\Vert \sum_{i=1}^{k} X_{i} \Biggr\Vert >\epsilon2^{(l-1) \alpha}+y \Biggr)\,dy \\& \quad \leq C\sum_{n=1}^{\infty}n^{\alpha r-2-\alpha} \int_{0}^{\infty}P \Biggl( \max_{1\leq k\leq n} \Biggl\Vert \sum_{i=1}^{k} X_{i} \Biggr\Vert >\epsilon n^{\alpha}2^{- \alpha}+y \Biggr) \,dy \\& \quad = C\sum_{n=1}^{\infty}n^{\alpha r-2-\alpha}E \Biggl( \max_{1\leq k\leq n} \Biggl\Vert \sum_{i=1}^{k} X_{i} \Biggr\Vert -\epsilon^{\prime} n^{\alpha } \Biggr)^{+}< \infty \quad{ \bigl(\text{by (3.2)} \bigr)}, \end{aligned}$$

where $\epsilon^{\prime}=\epsilon2^{-\alpha}$. Hence the proof (3.4) is completed. □

### Corollary 3.4

*Let* *r* *and* *α* *be positive real numbers such that* $1\leq r<2$, $\alpha r> 1$ *and* $\alpha>\frac{1}{2}$. *Let* $\{X_{n}, n\geq1\}$ *be a sequence of* *H*-*valued CNA random vectors with zero means*. *If* $\{X_{n}, n\geq1\}$ *is coordinatewise weakly upper bounded by a random vector* *X*, *then* (2.2) *implies*

3.5 $$ \sum_{n=1}^{\infty}n^{\alpha r-2}P \Biggl(\sup _{k\geq n}k^{-\alpha} \Biggl\Vert \sum _{i=1}^{k} X_{i} \Biggr\Vert >\epsilon \Biggr)< \infty \quad{\textit{for all }} \epsilon>0. $$

### Proof

Inspired by the proof of Theorem 12.1 of Gut [19], we can prove it and omit the proof. □

The following theorem shows that complete convergence and complete moment convergence still hold under a sequence of identically distributed *H*-valued CNA random vectors with zero means.

### Theorem 3.5

*Let* *r* *and* *α* *be positive real numbers such that* $1\leq r<2$ *and* $\alpha r> 1$. *Let* $\{X_{n}, n\geq1\}$ *be a sequence of* *H*-*valued CNA random vectors with zero means*. *Assume that* $\{X_{n}, n\geq1\}$ *are identically distributed random vectors with* (2.2′) *in Lemma* 2.6. *Then* (3.1), (3.4) *and* (3.5) *hold*.

### Proof

The proofs are similar to those of Theorem 3.1, Theorem 3.3 and Corollary 3.4, respectively. □

### Theorem 3.6

*Let* *r* *and* *α* *be positive real numbers such that* $\alpha r \geq1$ *and* $\{X_{n}, n\geq1\}$ *be a sequence of* *H*-*valued CNA random vectors with zero means*. *If* $\{X_{n}, n\geq1\}$ *is coordinatewise weakly bounded by a random vector* *X* *satisfying* (2.11) *and* (2.12), *then* (3.1) *holds*.

### Proof

By Lemma 2.7 and Theorem 3.1 the result follows. □

## Conclusions

1. In Section 3 we have obtained the complete moment convergence for a sequence of mean zero *H*-valued CNA random vectors which is coordinatewise weakly upper bonded by a random variable and the related results.
2. Theorem 3.1 generalizes the complete convergence for a sequence of mean zero *H*-valued CNA random vectors in Huan et al. [2] to the complete moment convergence.
